# Supplementary material for: Use of a Longer Aglycon Moiety Bearing Sialyl α(2→3) Lactoside on the Glycopolymer for Lectin Evaluation
Source: Polymers (Basel). 2023 Feb 17;15(4):998. doi: 10.3390/polym15040998 (PMC9959589; doi:10.3390/polym15040998)
Supplement: Supplementary file 1 [file polymers-15-00998-s001.zip › polymers-2220041-supplementary.pdf]

Use of longer aglycon bearing with sialyl α(2→3) lactoside on the glycopolymer for lectin evaluation

Ryota Adachi[1], Takahiko Matsushita[1,2,3], Tetsuo Koyama[1], Ken Hatano[1,2,3], and Koji Matsuoka[1,2,3,*]

1 Area for Molecular Function, Division of Material Science, Graduate School of Science and Engineering, Saitama University, Sakura, Saitama 338-8570, Japan; r.adachi.764@ms.saimata-u.ac.jp (R.A); takahiko@fms.saitama-u.ac.jp (T.M.); koyama@fms.saitama-u.ac.jp (T.K.); khatano@fms.saitama-u.ac.jp (K.H)

2 Medical Innovation Research Unit (MiU), Advanced Institute of Innovative Technology (AIIT), Saitama University, Sakura, Saitama 338-8570, Japan

3 Area for Health Science, Strategic Research Center, Saitama University, Sakura, Saitama 338-8570, Japan

* Correspondence:koji@fms.saitama-u.ac.jp; Tel.: +81-7088194601 (K.M.)

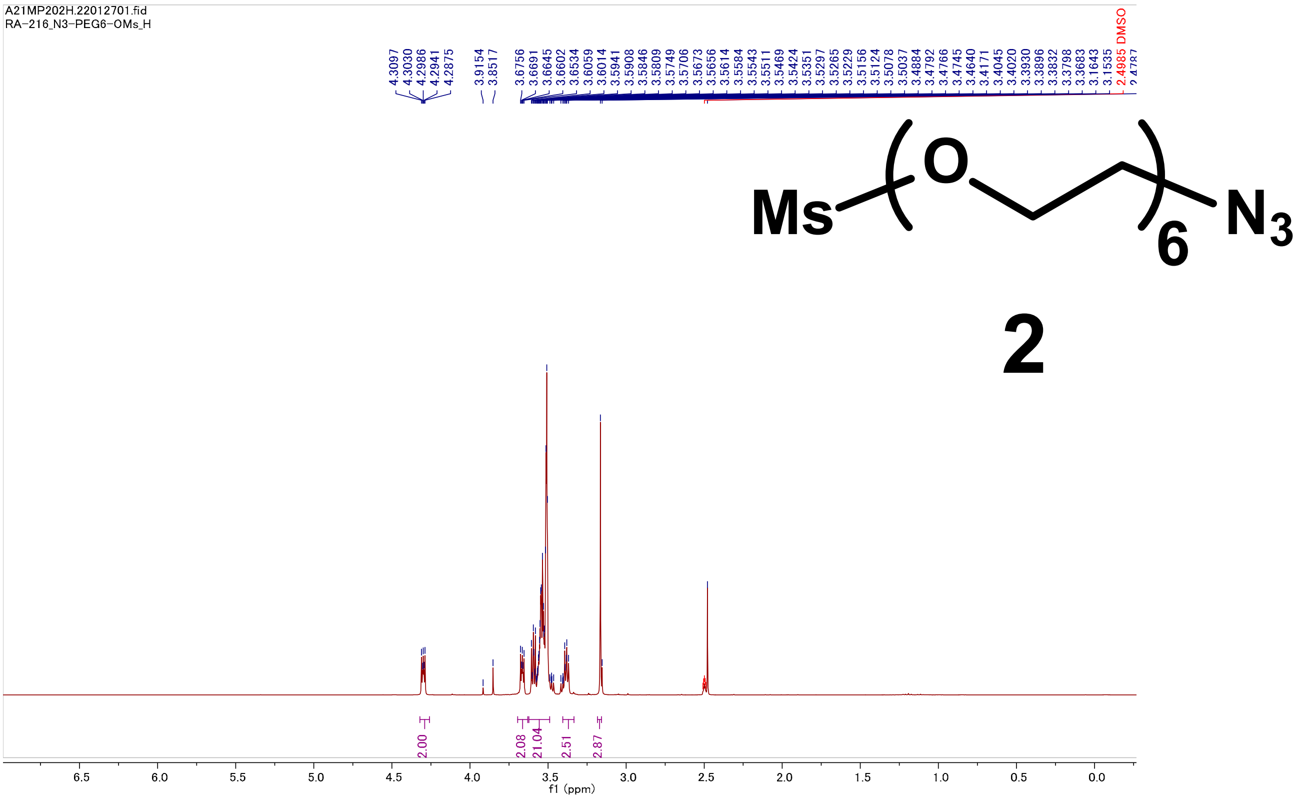

Figure S1. ^{1}H NMR spectrum of 17-azido-3,6,9,12,15-pentaoxaheptadecyl methanesulfonate (**2**) in DMSO-d_6

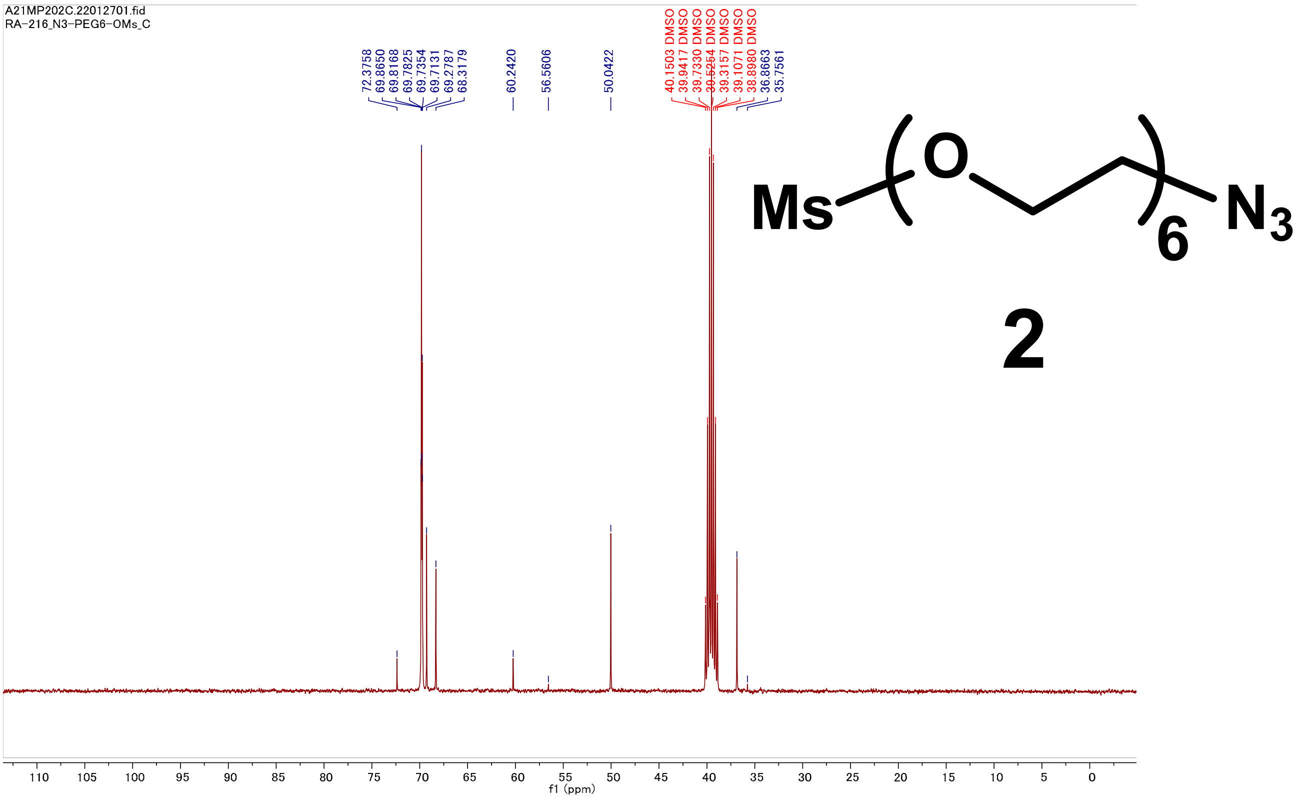

Figure S2. ^{13}C NMR spectrum of 17-azido-3,6,9,12,15-pentaoxaheptadecyl methanesulfonate (**2**) in DMSO-d_6

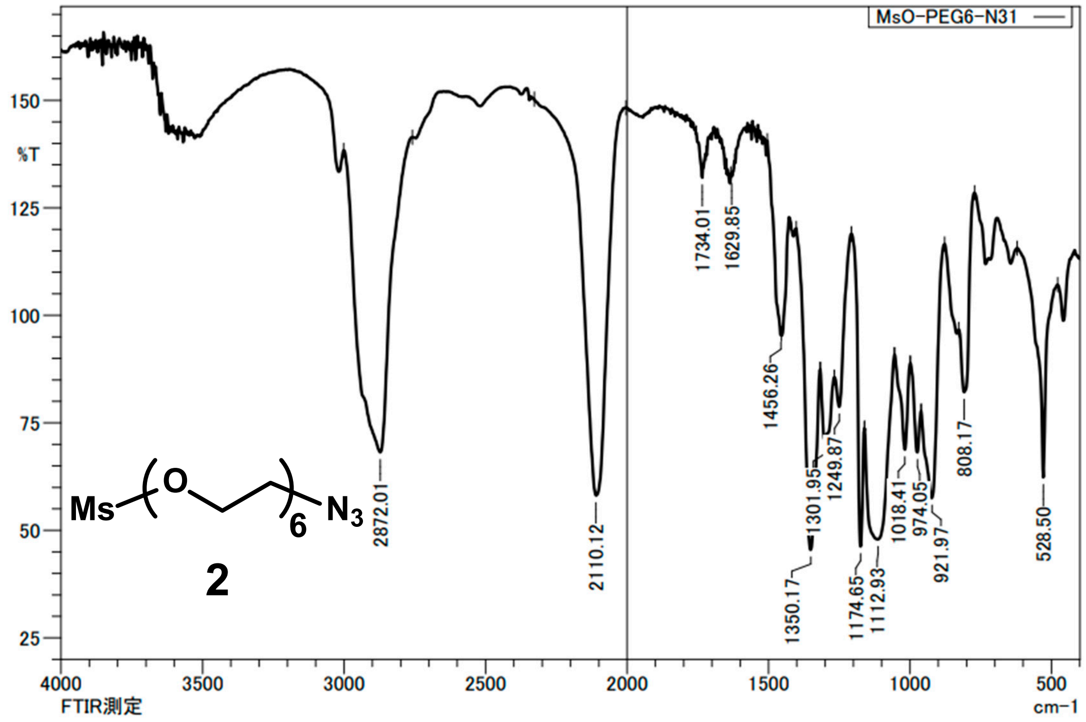

Figure S3. IR spectrum of 17-azido-3,6,9,12,15-pentaoxaheptadecyl methanesulfonate (**2**)

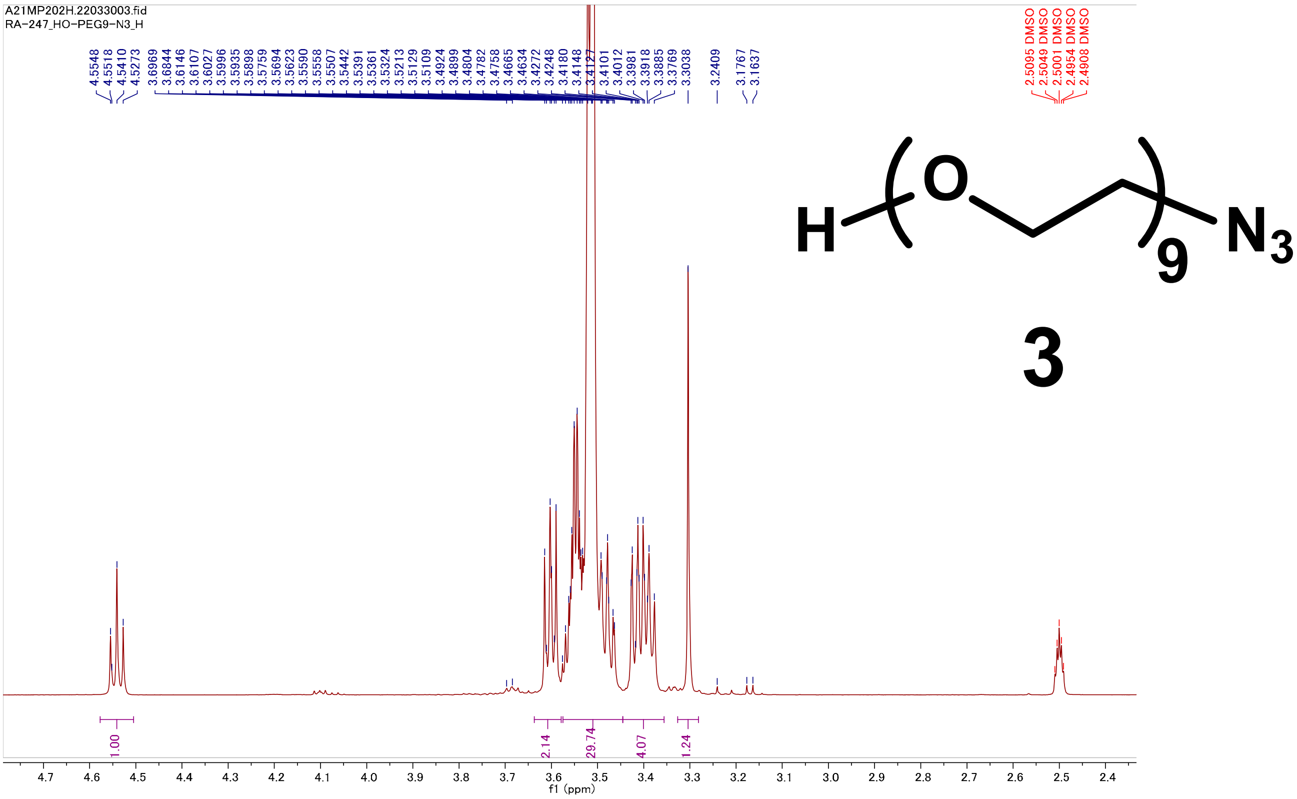

Figure S4. ^{1}H NMR spectrum of 26-azido-3,6,9,12,15,18,21,24-octaoxahexacosan-1-ol (**3**) in DMSO-d_6

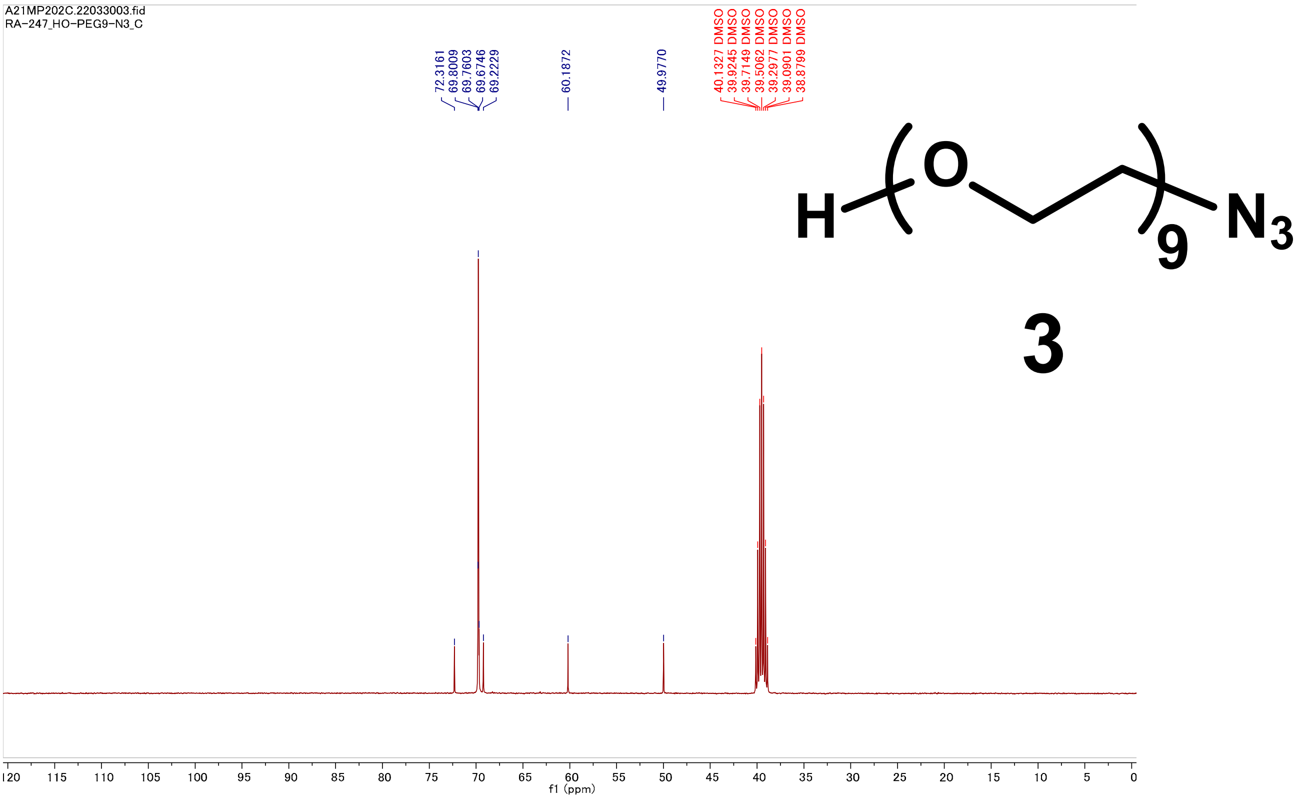

Figure S5. ^{13}C NMR spectrum of 26-azido-3,6,9,12,15,18,21,24-octaoxahexacosan-1-ol (**3**) in DMSO-d_6

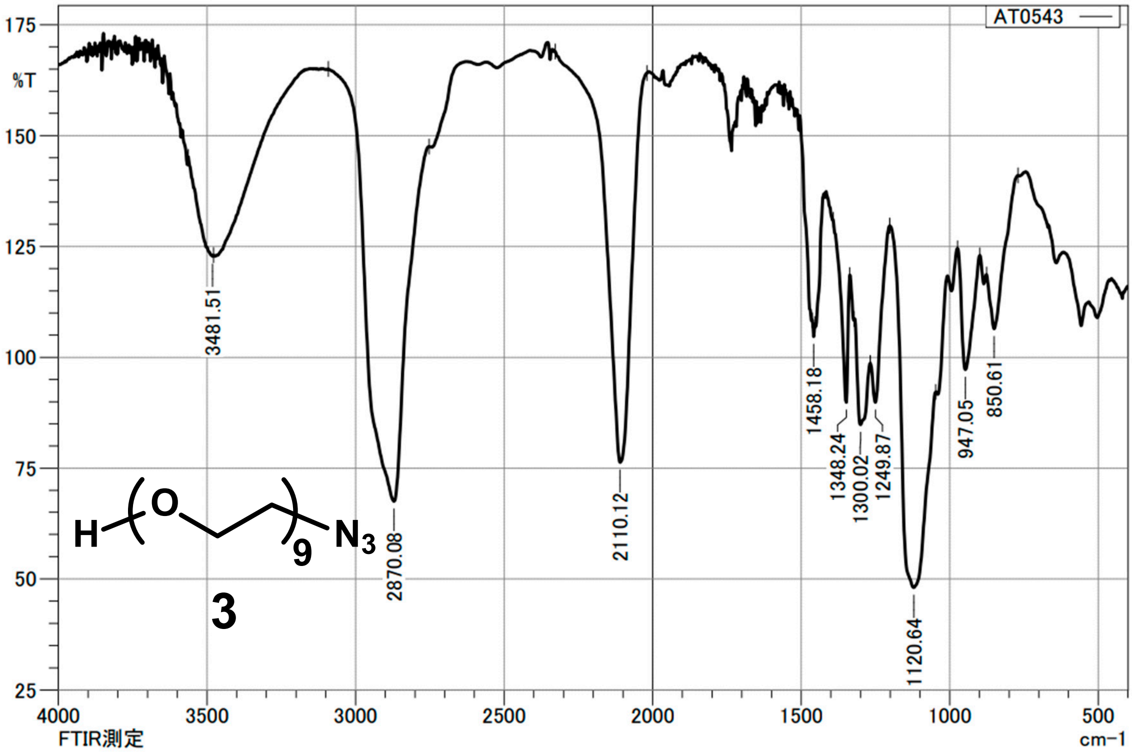

Figure S6. IR spectrum of 26-azido-3,6,9,12,15,18,21,24-octaoxahexacosan-1-ol (**3**)

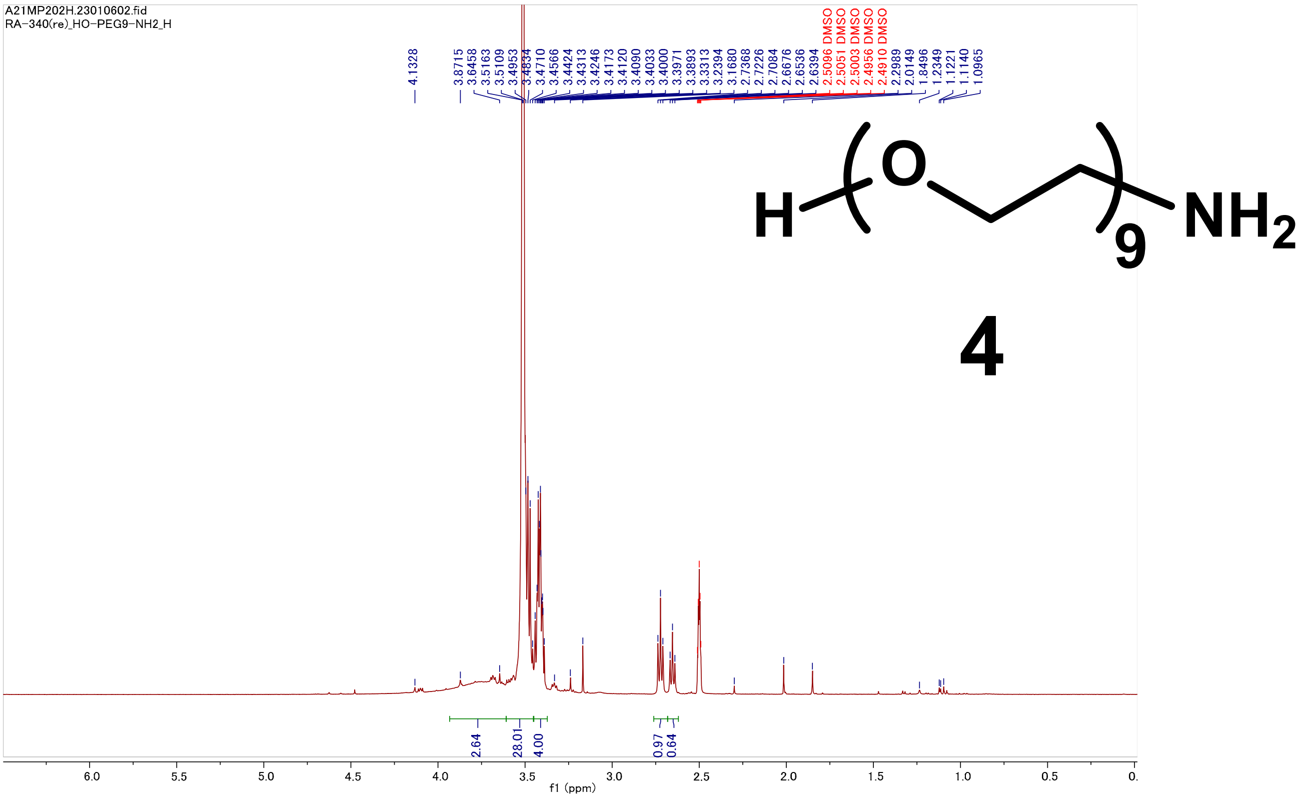

Figure S7. ^{1}H NMR spectrum of 26-amino-3,6,9,12,15,18,21,24-octaoxahexacosan-1-ol (**4**) in DMSO-d_6

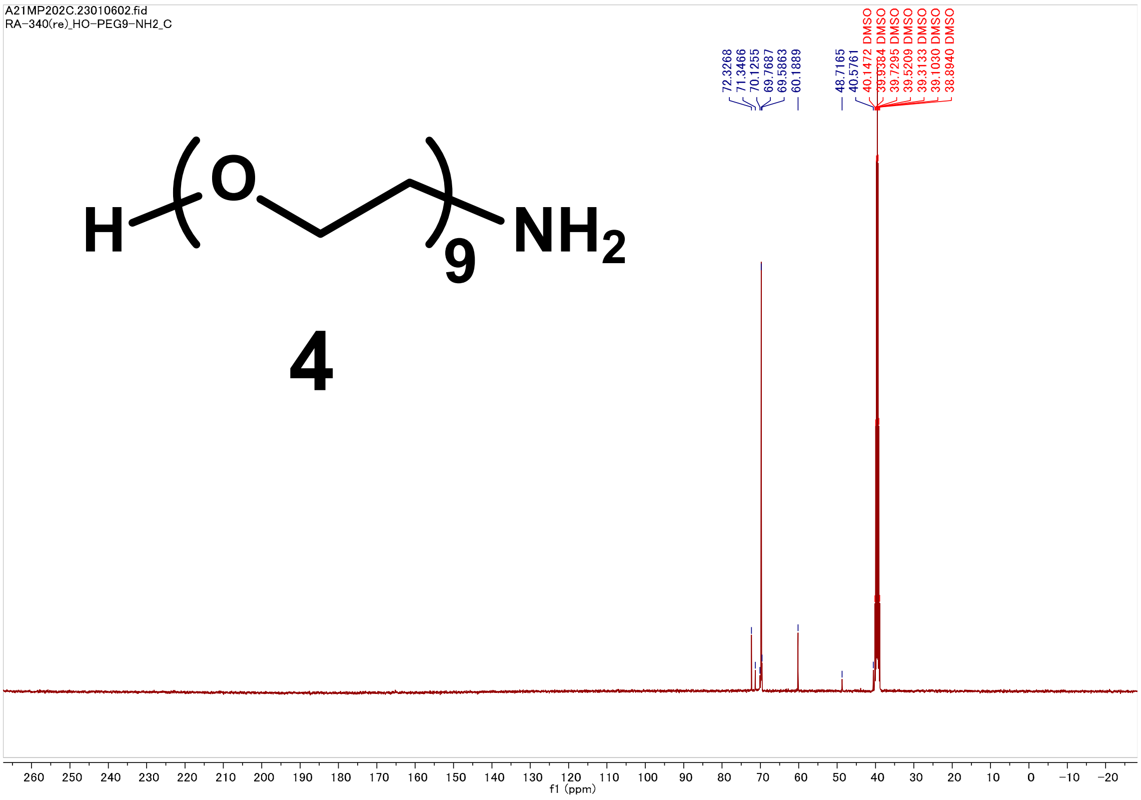

Figure S8. ^{13}C NMR spectrum of 26-amino-3,6,9,12,15,18,21,24-octaoxahexacosan-1-ol (**4**) in DMSO-d_6

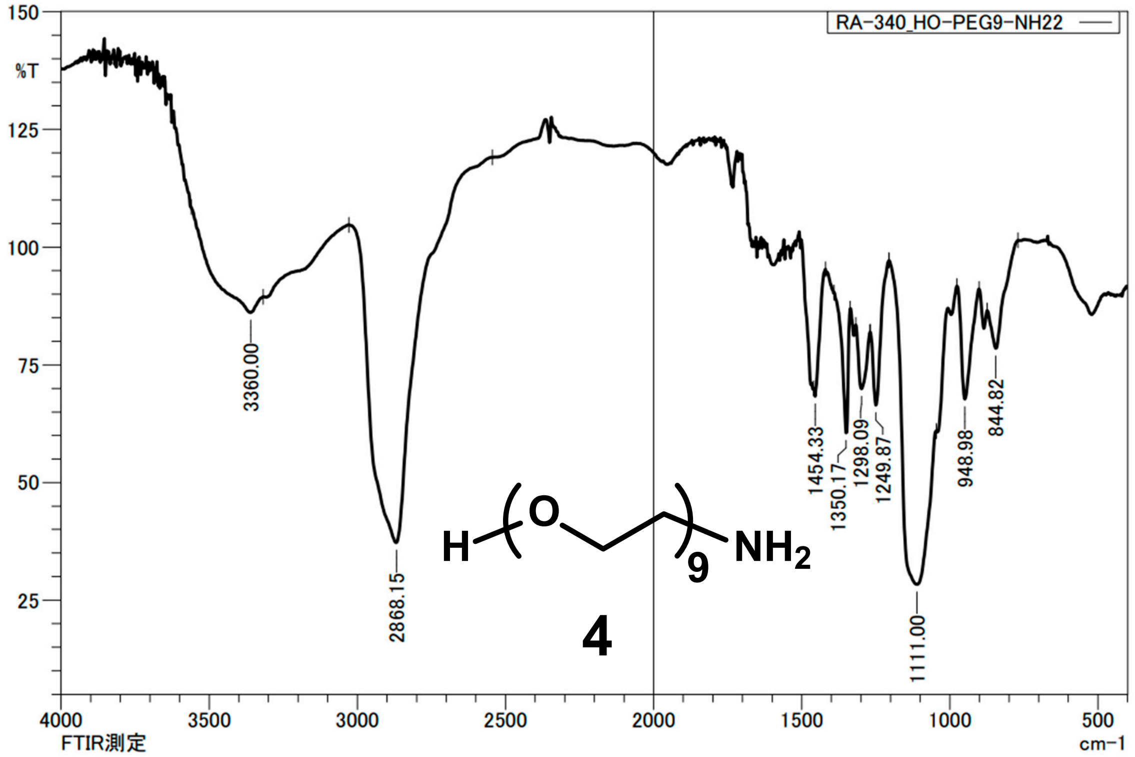

Figure S9. IR spectrum of 26-amino-3,6,9,12,15,18,21,24-octaoxahexacosan-1-ol (**4**)

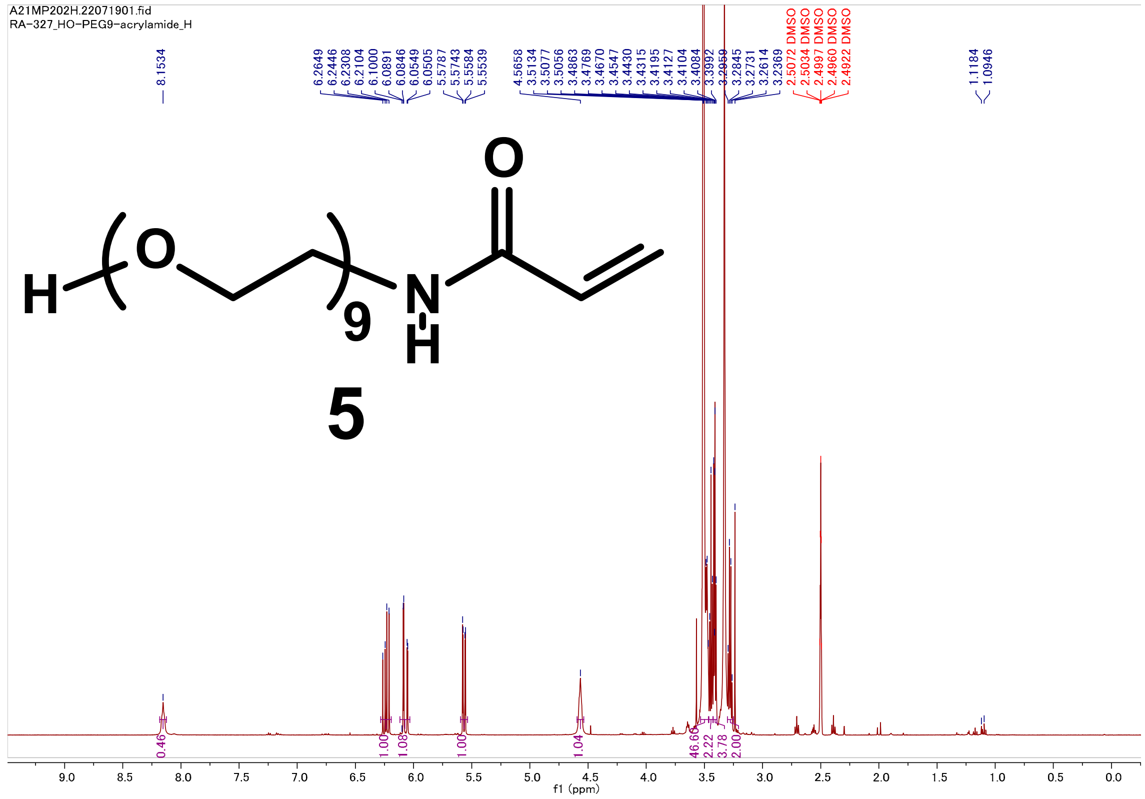

Figure S10. ^{1}H NMR spectrum of *N*-(26-hydroxy-3,6,9,12,15,18,21,24-octaoxahexacosyl)acrylamide (**5**) in DMSO-d_6

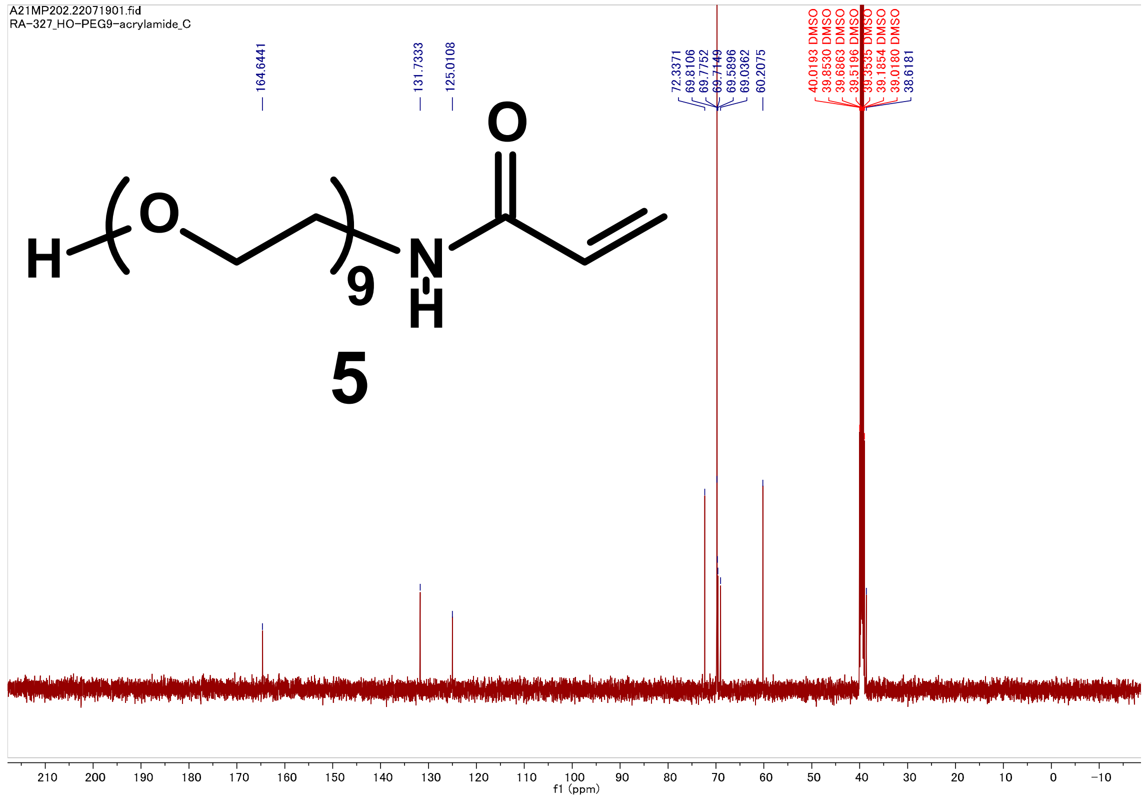

Figure S11. ^{13}C NMR spectrum of *N*-(26-hydroxy-3,6,9,12,15,18,21,24-octaoxahexacosyl)acrylamide (**5**) in DMSO-d_6

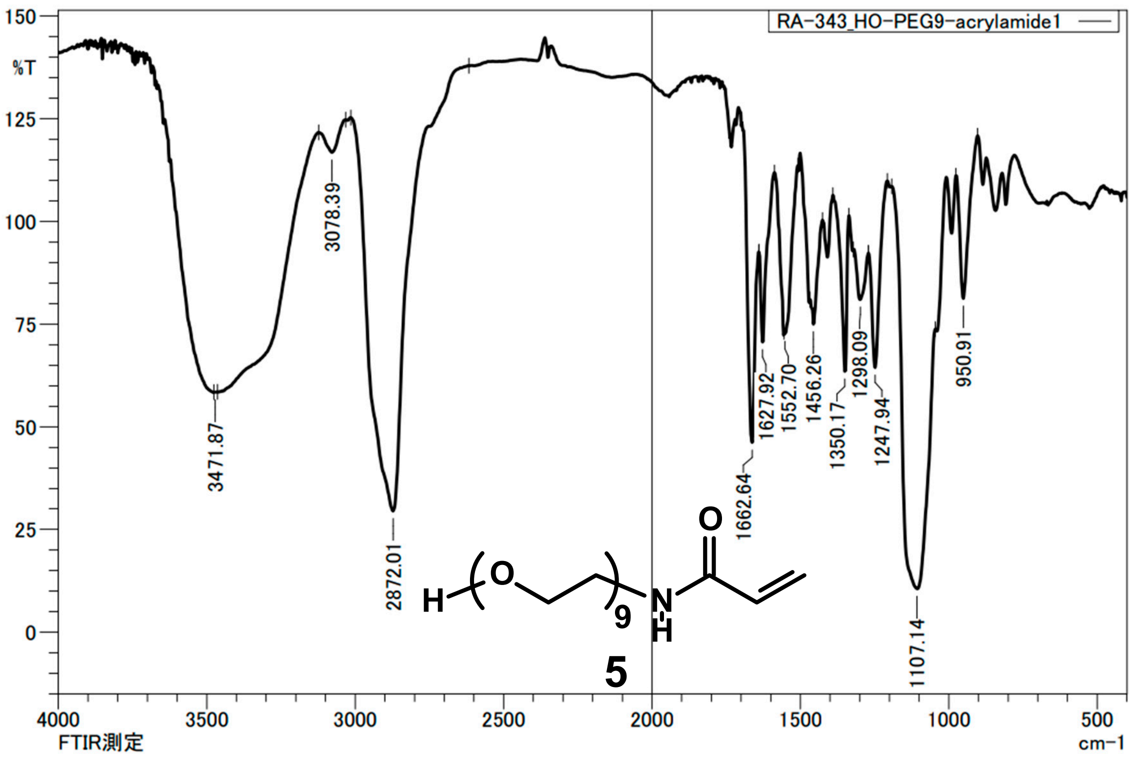

Figure S12. IR spectrum of *N*-(26-hydroxy-3,6,9,12,15,18,21,24-octaoxahexacosyl)acrylamide (**5**)

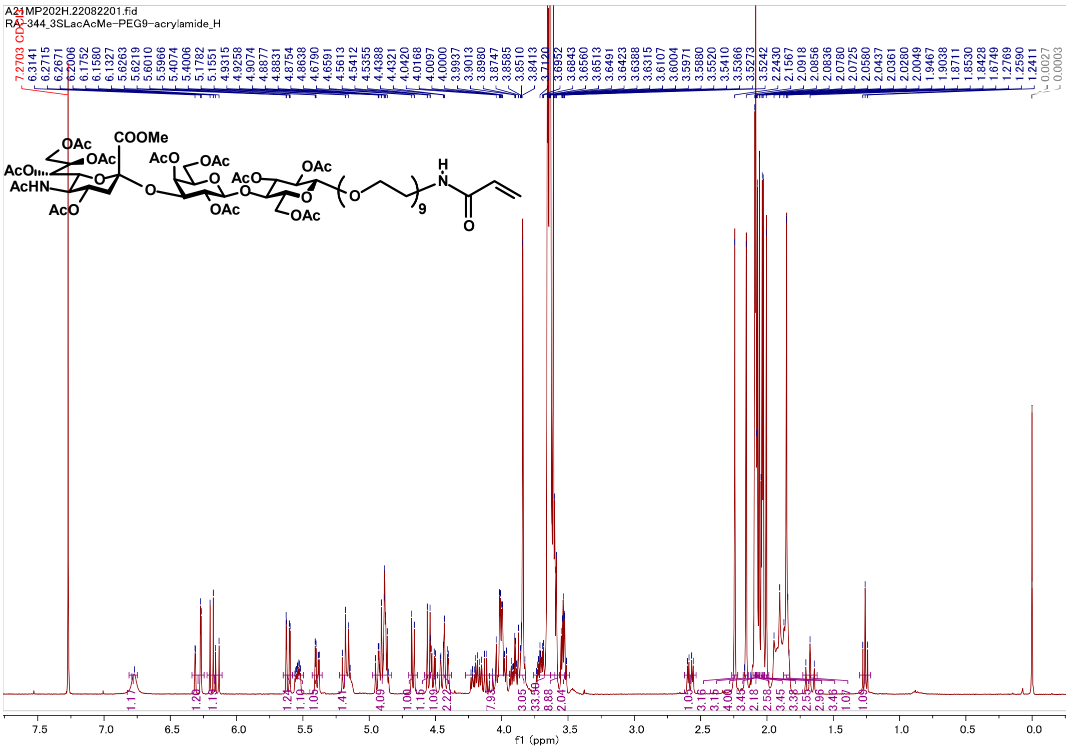

Figure S13. ^{1}H NMR spectrum of 28-oxo-3,6,9,12,15,18,21,24-octaoxa-27-azatriacont-29-en-1-yl [Methyl(5-acetamido-4,7,8,9-tetra-*O*-acetyl-3,5-dideoxy-D̲-glycero-α-D̲-galacto-2-nonulopyranosyl)onate]-(2→3)-*O*-(2,4,6-tri-*O*-acetyl-β-D̲-galactopyranosyl)-(1→4)-2,3,6-tri-*O*-acetyl-β-D̲-glucopyranoside (**7**) in $CDCl_3$

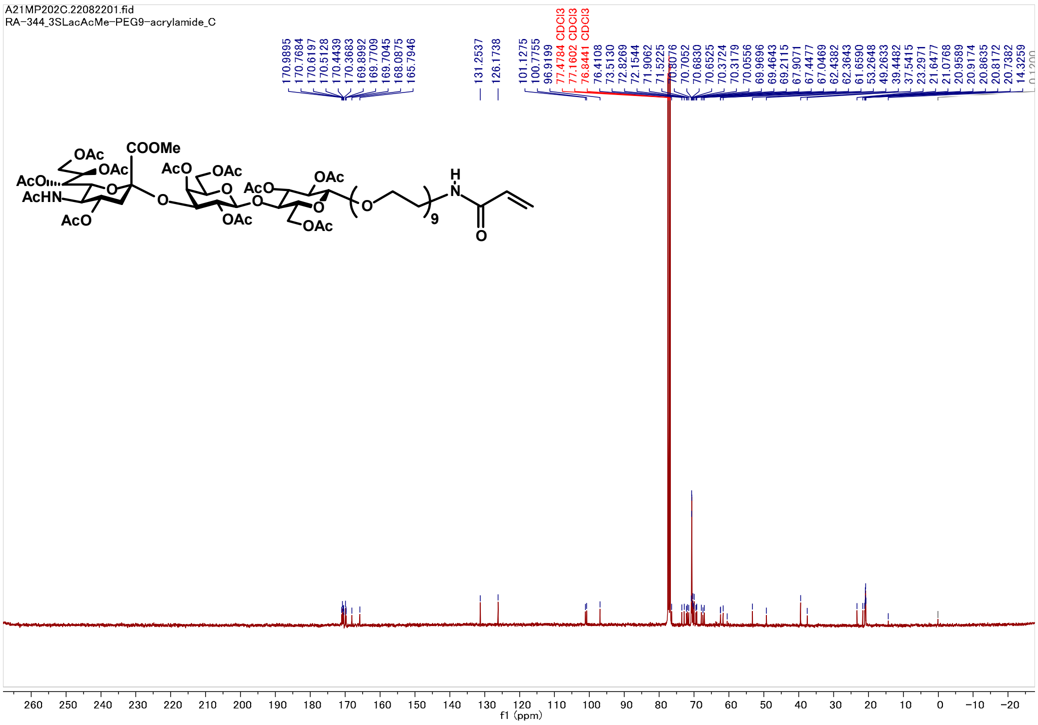

Figure S14. ^{13}C NMR spectrum of 28-oxo-3,6,9,12,15,18,21,24-octaoxa-27-azatriacont-29-en-1-yl [Methyl(5-acetamido-4,7,8,9-tetra-*O*-acetyl-3,5-dideoxy-D̲-glycero-α-D̲-galacto-2-nonulopyranosyl)onate]-(2→3)-*O*-(2,4,6-tri-*O*-acetyl-β-D̲-galactopyranosyl)-(1→4)-2,3,6-tri-*O*-acetyl-β-D̲-glucopyranoside (**7**) in $CDCl_3$

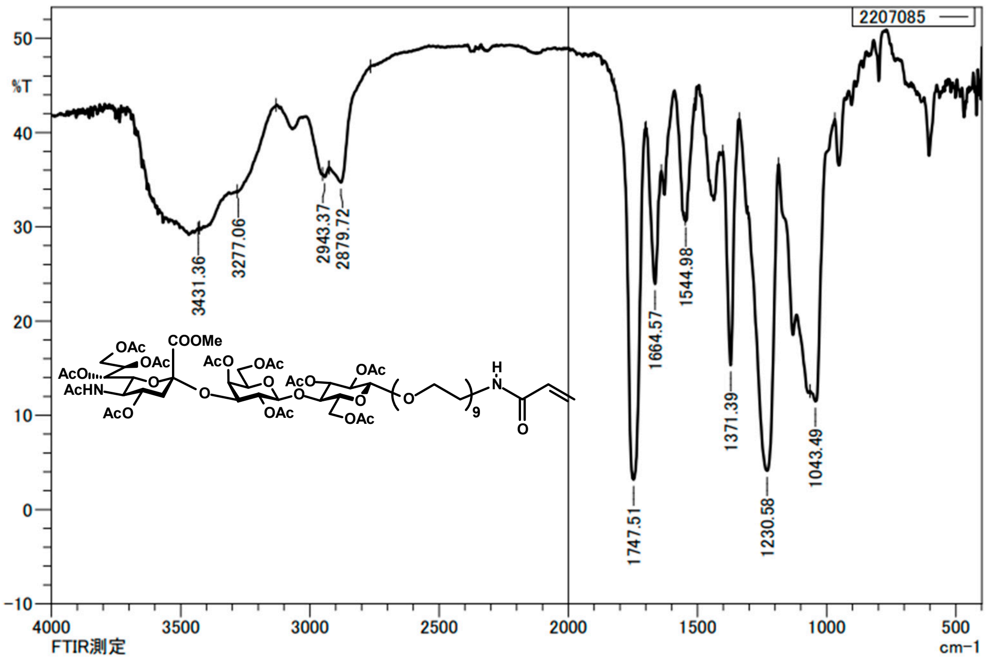

Figure S15. IR spectrum of 28-oxo-3,6,9,12,15,18,21,24-octaoxa-27-azatriacont-29-en-1-yl [Methyl(5-acetamido-4,7,8,9-tetra-*O*-acetyl-3,5-dideoxy-D-glycero-α-D-galacto-2-nonulopyranosyl)onate]-(2→3)-*O*-(2,4,6-tri-*O*-acetyl-β-D-galactopyranosyl)-(1→4)-2,3,6-tri-*O*-acetyl-β-D-glucopyranoside (**7**)

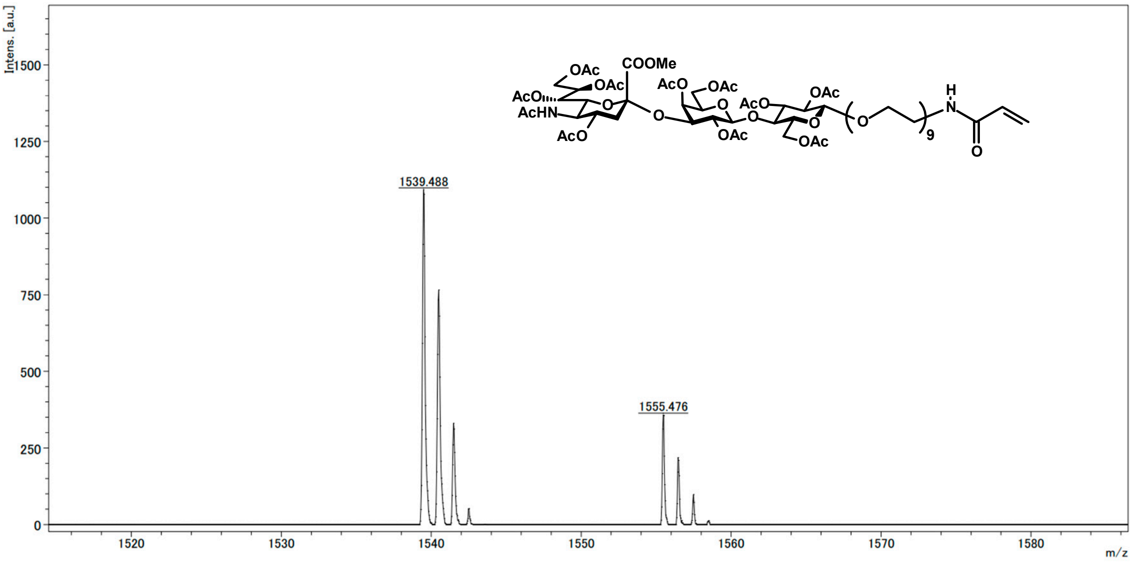

Figure S16. MALDI-TOF MS spectrum of 28-oxo-3,6,9,12,15,18,21,24-octaoxa-27-azatriacont-29-en-1-yl [Methyl(5-acetamido-4,7,8,9-tetra-*O*-acetyl-3,5-dideoxy-D-glycero-α-D-galacto-2-nonulopyranosyl)onate]-(2→3)-*O*-(2,4,6-tri-*O*-acetyl-β-D-galactopyranosyl)-(1→4)-2,3,6-tri-*O*-acetyl-β-D-glucopyranoside (**7**)

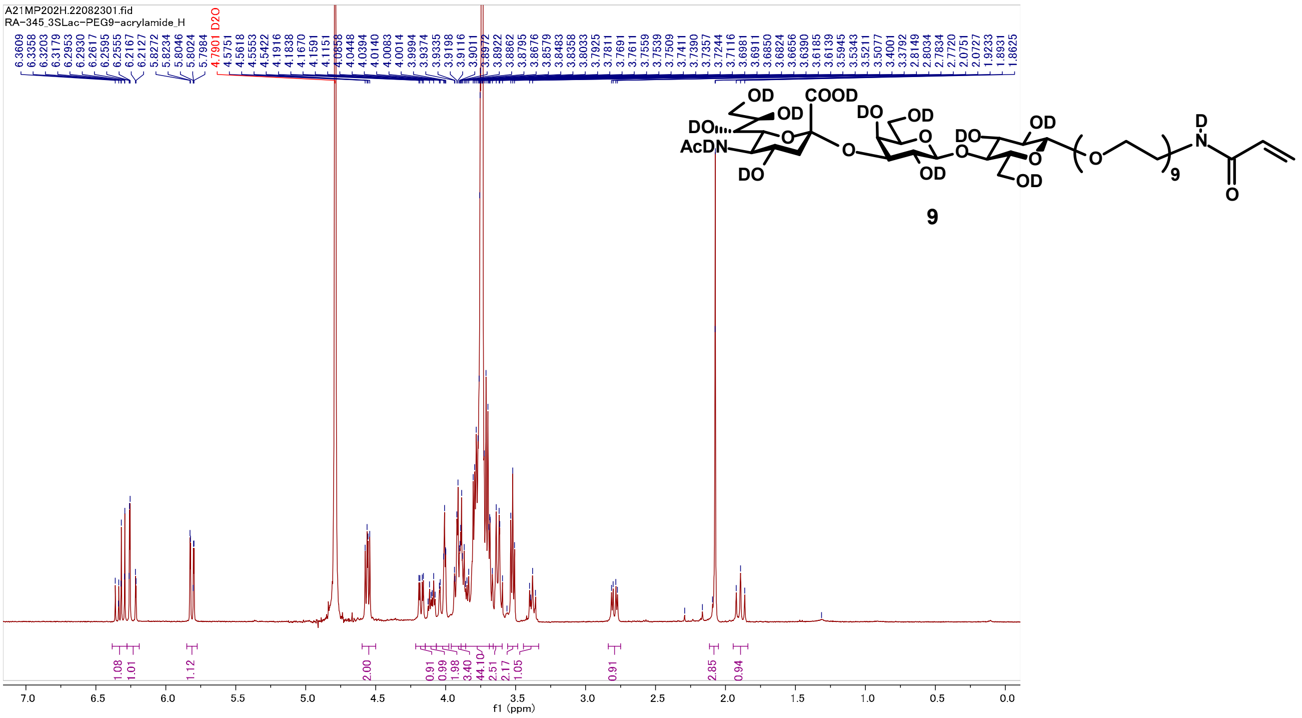

Figure S17. ^{1}H NMR spectrum of 28-oxo-3,6,9,12,15,18,21,24-octaoxa-27-azatriacont-29-en-1-yl (5-acetamido-3,5-dideoxy-D-glycero-α-D-galacto-2-nonulopyranosyl)-(2→3)-*O*-(β-D-galactopyranosyl)-(1→4)-β-D-glucopyranoside (**7**) in D_2O

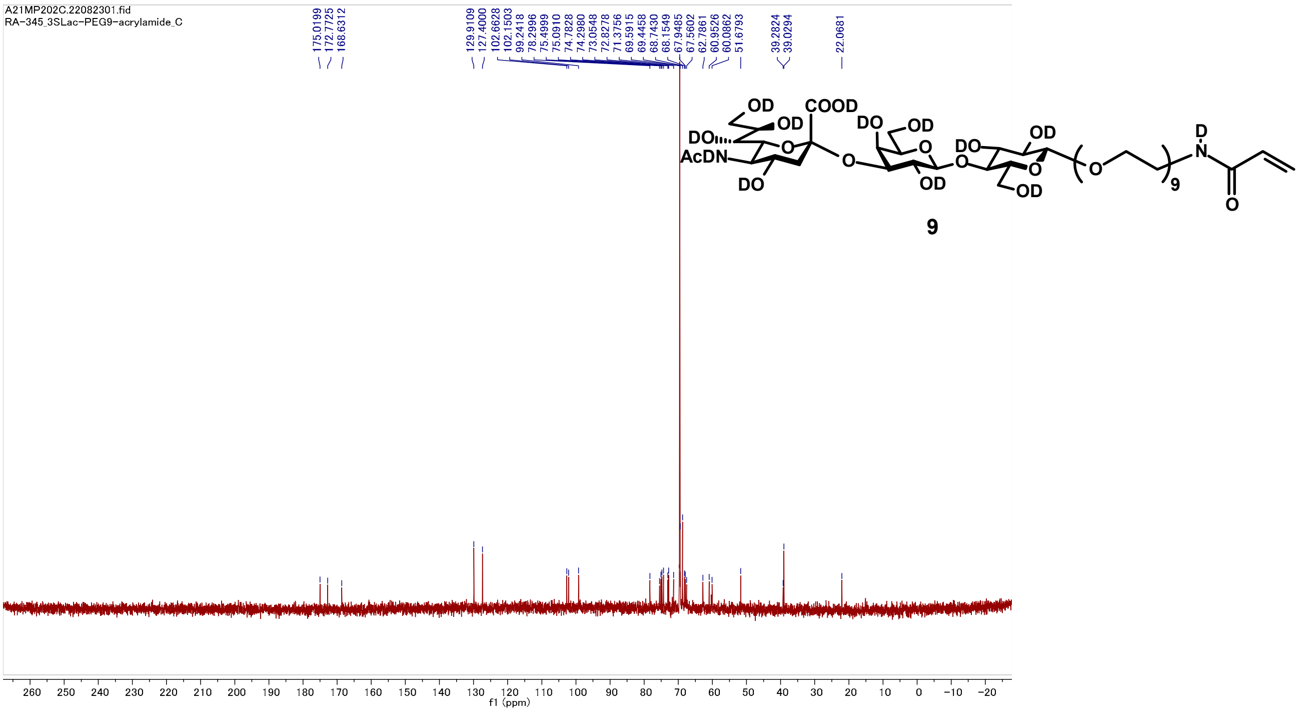

Figure S18. ^{13}C NMR spectrum of 28-oxo-3,6,9,12,15,18,21,24-octaoxa-27-azatriacont-29-en-1-yl (5-acetamido-3,5-dideoxy-D-glycero-α-D-galacto-2-nonulopyranosyl)-(2→3)-*O*-(β-D-galactopyranosyl)-(1→4)-β-D-glucopyranoside (**7**) in D_2O

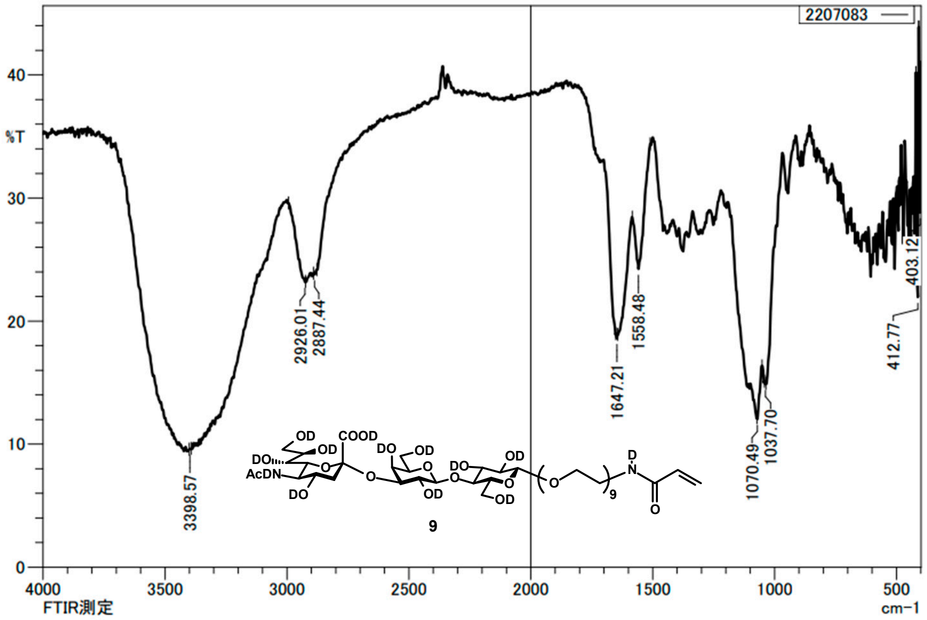

Figure S19. IR spectrum of 28-oxo-3,6,9,12,15,18,21,24-octaoxa-27-azatriacont-29-en-1-yl (5-acetamido-3,5-dideoxy-D-glycero-α-D-galacto-2-nonulopyranosyl)-(2→3)-*O*-(β-D-galactopyranosyl)-(1→4)-β-D-glucopyranoside (**7**)

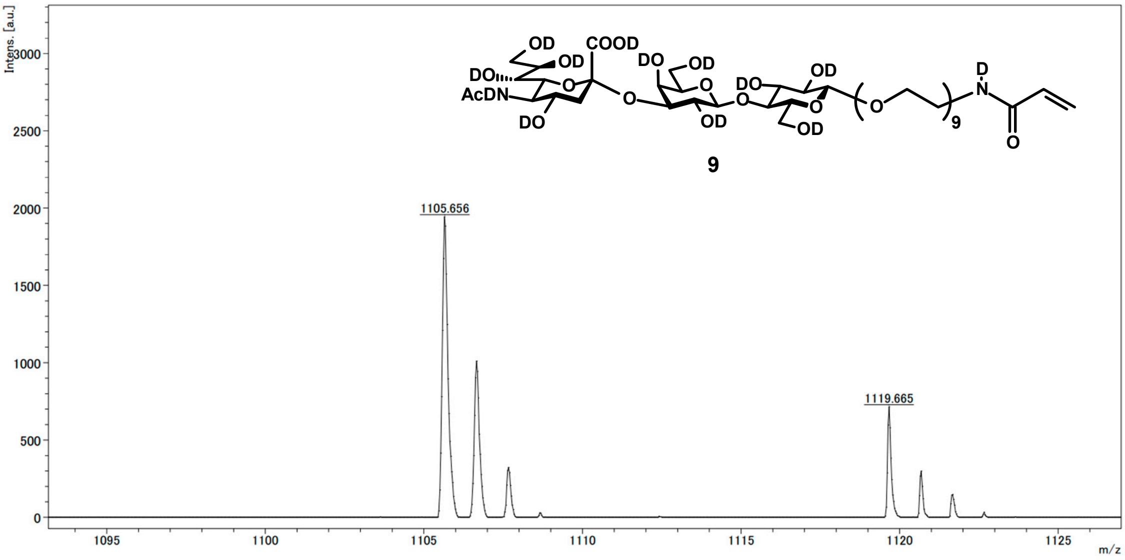

Figure S20. MALDI-TOF MS spectrum of 28-oxo-3,6,9,12,15,18,21,24-octaoxa-27-azatriacont-29-en-1-yl (5-acetamido-3,5-dideoxy-D-glycero-α-D-galacto-2-nonulopyranosyl)-(2→3)-*O*-(β-D-galactopyranosyl)-(1→4)-β-D-glucopyranoside (**7**)

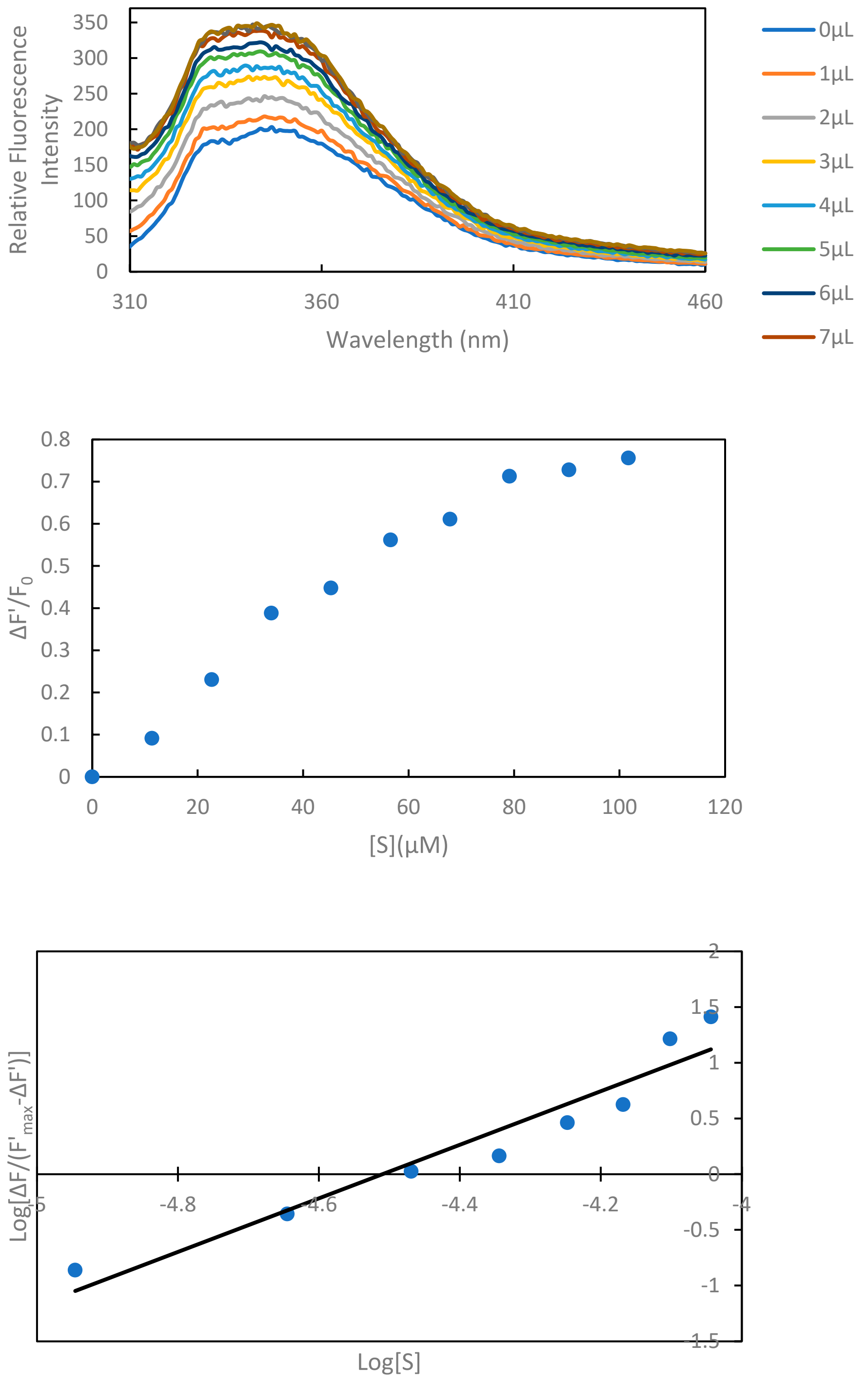

Figure S21. Biological evaluations of the WGA—carbohydrate interaction using glycomonomer **9**. (Top) Changes in fluorescence emission spectra of WGA (0.65 μM, 3.0 mL, 50 mM Tris-HCl buffer containing 1.25 M NaCl and 25 mM $CaCl_2$, pH 7.5, 4 ± 0.1 °C). (Middle) Plots of $\Delta F'/F_0$ versus [S], where $\Delta F'$ is change in the intensity at 348 nm of WGA with various concentrations, F_0 is the intensity of WGA alone, and [S] is the total ligand concentration based on the sugar residue concentration. (Bottom) Hill plots of log $[\Delta F'/(\Delta F'_{MAX}-\Delta F')]$ versus log [S].

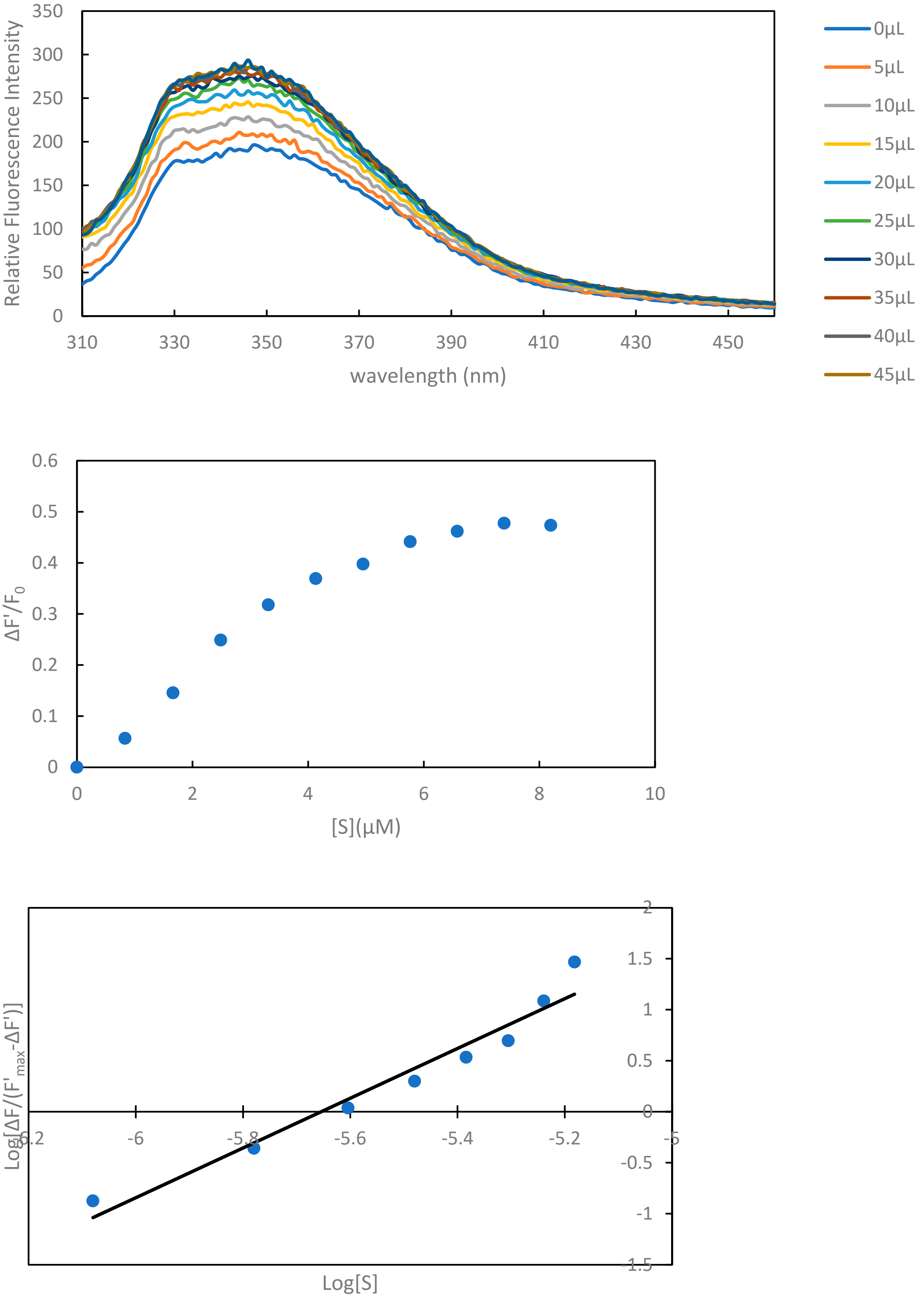

Figure S22. Biological evaluations of the WGA—carbohydrate interaction using homopolymer **10a**. (Top) Changes in fluorescence emission spectra of WGA (0.65 μM, 3.0 mL, 50 mM Tris-HCl buffer containing 1.25 M NaCl and 25 mM $CaCl_2$, pH 7.5, 4 ± 0.1 °C). (Middle) Plots of $\Delta F'/F_0$ versus [S], where $\Delta F'$ is change in the intensity at 348 nm of WGA with various concentrations, F_0 is the intensity of WGA alone, and [S] is the total ligand concentration based on the sugar residue concentration. (Bottom) Hill plots of log $[\Delta F'/(\Delta F'_{MAX}\text{-}\Delta F')]$ versus log [S].

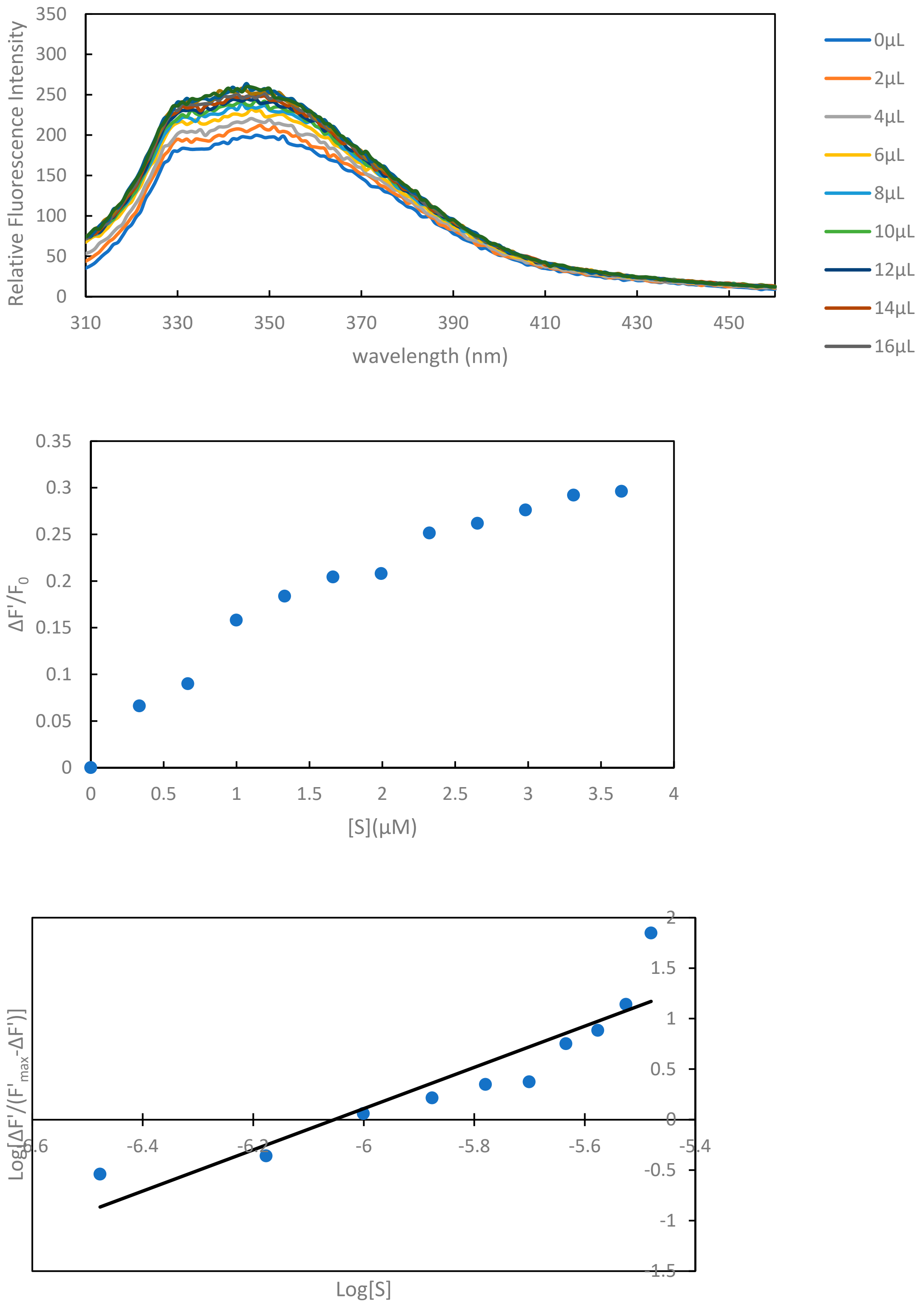

Figure S23. Biological evaluations of the WGA—carbohydrate interaction using copolymer **10b**. (Top) Changes in fluorescence emission spectra of WGA (0.65 μM, 3.0 mL, 50 mM Tris-HCl buffer containing 1.25 M NaCl and 25 mM $CaCl_2$, pH 7.5, 4 ± 0.1 °C). (Middle) Plots of $\Delta F'/F_0$ versus [S], where $\Delta F'$ is change in the intensity at 348 nm of WGA with various concentrations, F_0 is the intensity of WGA alone, and [S] is the total ligand concentration based on the sugar residue concentration. (Bottom) Hill plots of log [$\Delta F'/(\Delta F'_{MAX}-\Delta F')$] versus log [S].

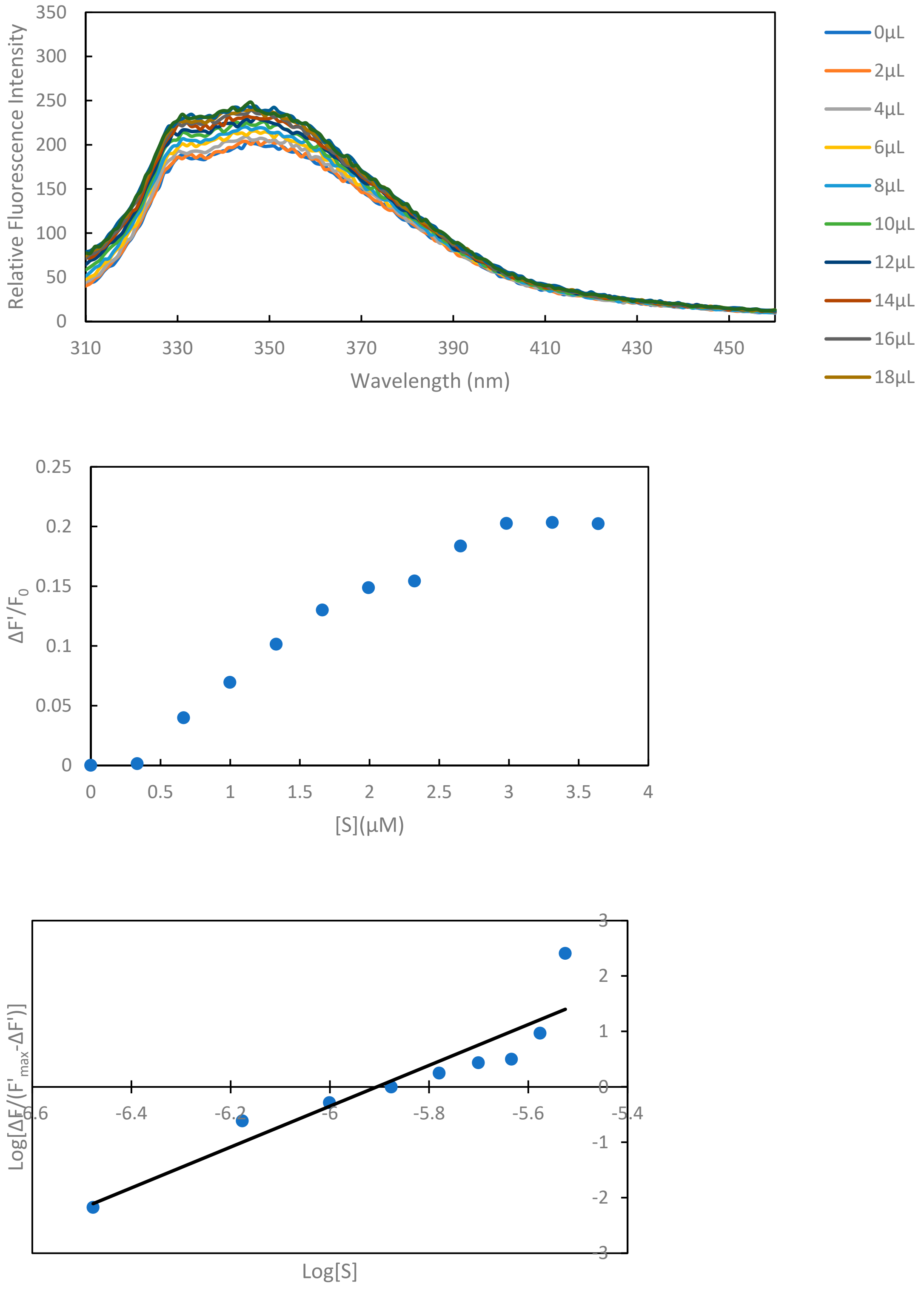

Figure S24. Biological evaluations of the WGA—carbohydrate interaction using copolymer **10c**. (Top) Changes in fluorescence emission spectra of WGA (0.65 μM, 3.0 mL, 50 mM Tris-HCl buffer containing 1.25 M NaCl and 25 mM $CaCl_2$, pH 7.5, 4 ± 0.1 °C). (Middle) Plots of $\Delta F'/F_0$ versus [S], where $\Delta F'$ is change in the intensity at 348 nm of WGA with various concentrations, F_0 is the intensity of WGA alone, and [S] is the total ligand concentration based on the sugar residue concentration. (Bottom) Hill plots of log [$\Delta F'/(\Delta F'_{MAX}-\Delta F')$] versus log [S].

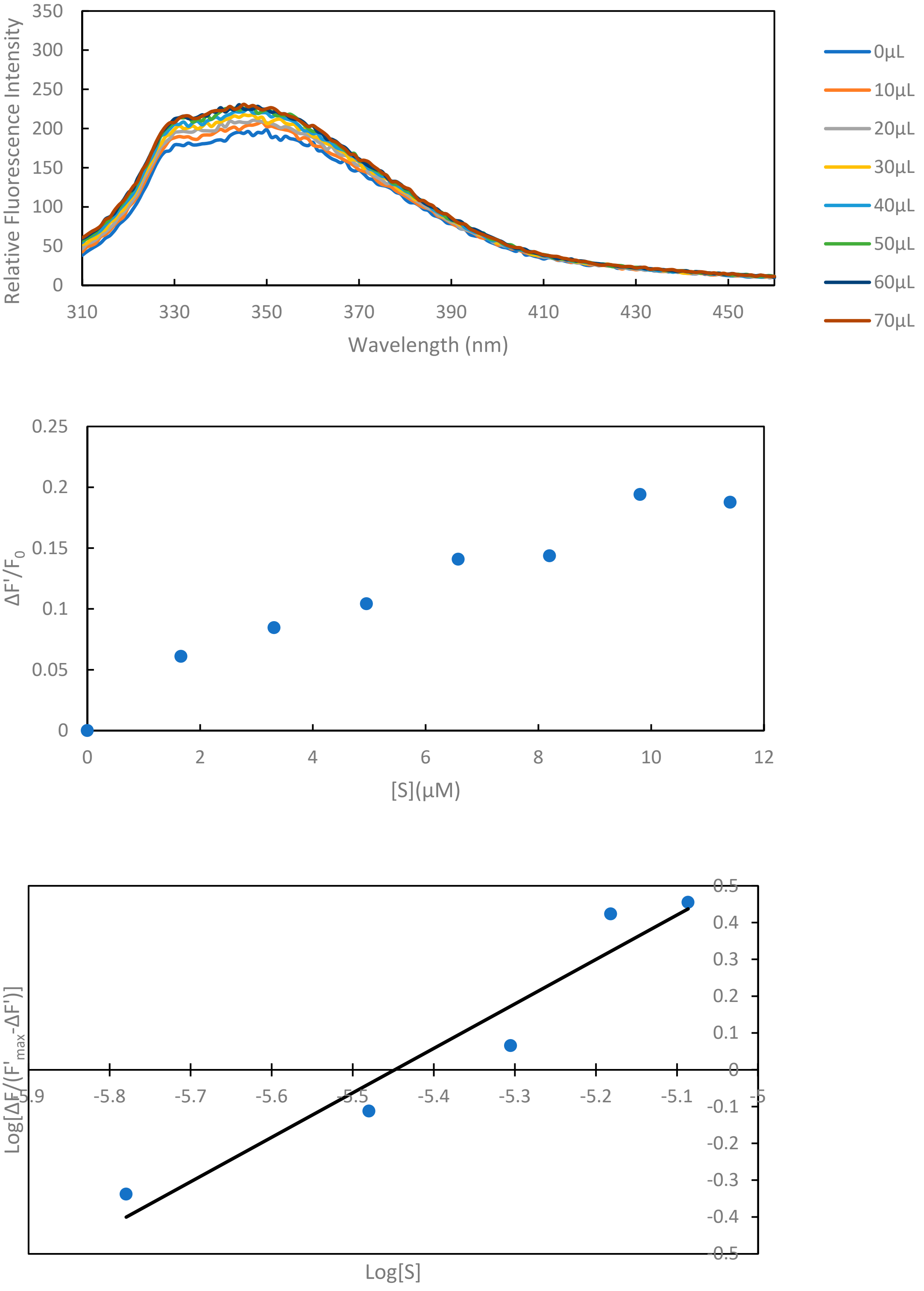

Figure S25. Biological evaluations of the WGA—carbohydrate interaction using copolymer **10d**. (Top) Changes in fluorescence emission spectra of WGA (0.65 μM, 3.0 mL, 50 mM Tris-HCl buffer containing 1.25 M NaCl and 25 mM $CaCl_2$, pH 7.5, 4 ± 0.1 °C). (Middle) Plots of $\Delta F'/F_0$ versus [S], where $\Delta F'$ is change in the intensity at 348 nm of WGA with various concentrations, F_0 is the intensity of WGA alone, and [S] is the total ligand concentration based on the sugar residue concentration. (Bottom) Hill plots of log [$\Delta F'/(\Delta F'_{MAX}-\Delta F')$] versus log [S].
